# A mechanoresponsive nano-sized carrier achieves intracellular release of drug on external ultrasound stimulus†

**DOI:** 10.1039/d2ra02307e

**Published:** 2022-06-06

**Authors:** Rosa Catania, David Onion, Emanuele Russo, Mischa Zelzer, Giuseppe Mantovani, Alan Huett, Snow Stolnik

**Affiliations:** School of Pharmacy, University of Nottingham Nottingham NG7 2RD UK snow.stolnik@nottingham.ac.uk; School of Life Sciences, University of Nottingham Nottingham NG7 2UH UK

## Abstract

Control over intracellular release of therapeutic compounds incorporated into nano-carriers will open new possibilities for targeted treatments of various diseases including cancer, and viral and bacterial infections. Here we report our study on mechanoresponsive nano-sized liposomes which, following internalization by cells, achieve intracellular delivery of encapsulated cargo on application of external ultrasound stimulus. This is demonstrated in a bespoke cell reporter system designed to assess free drug in cytoplasm. Biophysical analyses show that drug release is attributable to the action of a mechanoresponsive spiropyran-based compound embedded in the liposomal lipid membrane. Exposure to external ultrasound stimulus results in opening of the molecular structure of the embedded spiropyran, a consequent increase in liposomal lipid membrane fluidity, and size-dependent release of encapsulated model drugs, all pointing to lipid bilayer perturbation. The study hence illustrates feasibility of the proposed concept where intracellular drug release from mechanoresponsive liposomes can be triggered on demand by external ultrasound stimulus.

## Abstract 2

The study illustrates feasibility of the proposed concept where intracellular drug release from mechanoresponsive liposomes can be triggered on demand by external ultrasound stimulus.

## Graphical abstract

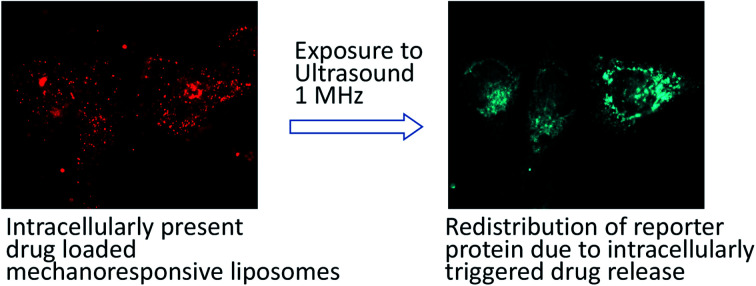

## Introduction

Ultrasound applied as an external stimulus offers potential for on demand triggered drug delivery from formulations intended for biomedical applications.^1^ Ultrasound is a non-invasive medical intervention and can be employed in a focused manner so that the energy applied to the surrounding non-targeted tissues is minimised.^2^ Different approaches have been studied and adopted, with the most advanced microbubble technology now moving towards different applications in clinical settings.^3,4^ There are different variations of this technology which, in essence, explore micron-size range bubbles which ‘explode’ on the application of ultrasound. This explosion damages the cytoplasmic membrane of nearby cells to facilitate entry into cells of drug molecules that are present in the vicinity within the extracellular environment.^5^ Microbubbles in a micron-size range are size optimised so that they explode when exposed to ultrasound of ∼1 MHz – frequency applicable in medical setting.^6^ In one recent example of this technology, gas-filled lipid microbubbles of approximately 6 μm were decorated with liposomes (of approximately 200 nm diameter and loaded with a drug) and shown to deliver into cells a membrane impermeant antibiotic (gentamicin) on applying ultrasound and disruption of cell plasma membrane.^7^ Another recent approach uses ultrasound as a stimulus to incite encapsulated sonosensitizer (protoporphyrin IX) to produce reactive oxygen species which then react with the liposomal membrane, causing damage and leading to the release into surrounding environment of a potent local anaesthetic, tetrodotoxin.^8^

Potential advantages of designing a sub-micron sized, *i.e.* nano-sized systems, relative to micron-sized microbubbles, arise from the fact that their size opens possibilities of systemic circulation and a consequent potential for targeting of, for instance, tissues with higher vascular permeability, such as cancer or inflammation. Furthermore, and importantly for this study, once they reached a target tissue, nano-sized systems offer a potential of accessing and delivery of cargo material intracellularly exploiting a natural process of endocytosis. A body of literature describes ultrasound-responsive formulations in the sub-micron size range, including as examples micelles,^9^ perfluorocarbon loaded liposomes or nanoemulsions,^10^ composites^11^ or hybrid^12^ mesoporous silica materials. These typically incorporate functionalities responsive to thermal or mechanical effects of ultrasound waves. In a ‘classical’ example, ThermoDox® liposomes, the lipid composition of liposomal membrane is optimised to create liposomes responsive to thermal effect caused by applied ultrasound.^13^

In this study we fabricated liposomes with size appropriate for endocytosis by non-phagocytic cells in tissues (*i.e.* normally in sub-300 nm diameter),^14^ whereby application of external ultrasound would cause disruptions of the liposomal lipid membrane, and a consequent intracellular cargo release, due to incorporation of a mechanoresponsive compound into the membrane; an approach that, to the best of our knowledge, has not been previously explored. Mechanoresponsive compounds are activated by application of mechanical force, whereby spiropyran-based mechanophore compounds are extensively studied.^15^ They are explored as probes which, inserted into a polymeric material, provide a molecular-scale reading of the local mechanical state, or with the view to transform materials' properties in response to the local mechanical environment. Considering ultrasound application, it has been demonstrated that mechanical force created by ultrasound, and consequent vibrations of polymeric chains to which mechanoresponsive molecules are attached, leads to changes in molecular structure of mechanoresponsive molecules and hence their physicochemical properties.^16^

**Fig. 1:**
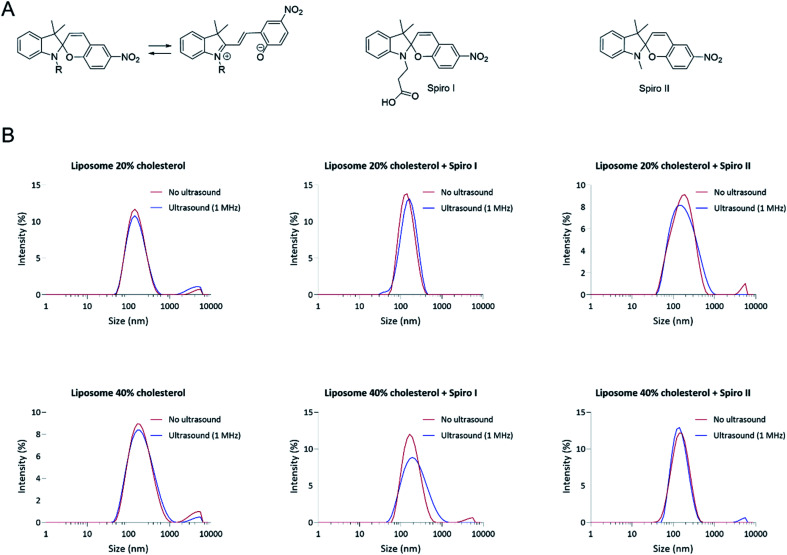
(A) Molecular structures of spiropyran mechanoresponsive molecules used in the fabrication of liposomes, denoted as spiro I and spiro II in the text, including their structure change to merocyanine form, and (B) particle size distribution profiles of fabricated liposomes. Hydrodynamic particle size distribution profiles (scattering intensity) of ‘control’ liposomes and liposomes containing 20 mol% mechanoresponsive compounds fabricated with either 20 (‘Liposome 20%) or 40 mol% (‘Liposome 40%’) of cholesterol in total lipid. Particle size distribution profiles shown prior (‘No ultrasound’) and following liposomes suspension exposure to ultrasound at 1 MHz for 6 minutes (‘Ultrasound’).

Here we demonstrate that a spiropyran-based compound (Fig. 1A) embedded into a liposomal lipid membrane undergoes a ring-opening to the merocyanine form on application of ultrasound and that consequent changes in the molecule's physicochemical properties (planarity, polarity and/or dipole moment)^16,17^ induce an increase in liposomal membrane fluidity, as judged from Laurdan generalised polarisation analysis. This increase in membrane fluidity/disorder results in a release of incorporated model drug (rapamycin) from liposomes internalised by cells, as illustrated in a cell reporter system developed for the study. Importantly, the cell culture studies indicate a differentiating effect, whereby ultrasound triggers a release of liposomes incorporated drug whilst, under the experimental conditions applied, cell morphology is not significantly altered, relative to untreated cells, to indicate negative effects on cells wellbeing.

## Experimental

### Synthesis of spiro I (3-(3′,3′-dimethyl-6-nitrospiro[chromene-2,2′-indolin]-1′-yl)propanoic acid)

Spiropyran I was prepared according to a previously reported procedure^18^ as follows. 3,3-Trimethyl-3*H*-indole (4.0 g, 25 mmol) and 3-iodopropanoic acid (5.4 g, 27 mmol) were mixed and left stirring at 100 °C under reflux for 3 hours. After cooling to ambient temperature, the obtained red solid product was dissolved in water (100 mL) and stirred for 12 hours. The solution was washed five times with chloroform (50 mL). The water phase was collected and the product obtained *via* lyophilisation (4.50 g, 12.5 mmol) was dissolved in 2-butanone (100 mL) in a round-bottom flask covered with aluminium foil. Piperidine (1.3 mL, 13.2 mmol) and 2-hydroxy-5-nitrobenzaldehyde (2.1 g, 12.56 mmol) were added and the red reaction mixture was heated to 100 °C and stirred under reflux for 3 hours. The reaction mixture was cooled to ambient temperature and stored for 12 hours without stirring at 0 °C. The precipitate was filtered and washed with methanol to obtain compound spiropyran I as a yellow powder (yield: 60%). ^1^H and COSY NMR spectra (Fig. S1†) were recorded on a Bruker DPX400 UltraShield™ Spectrometer and processed with MestReNova 14.1.2© 2020 Mestrelab Research S.L. All chemical shifts are reported in ppm (*δ*) relative to tetramethylsilane or referenced to the chemical shifts of residual solvent resonances. The following abbreviations are used to explain the multiplicities: s = singlet, d = doublet, dd = doublet od doublets, t = triplet, m = multiplet.

### ^1^H NMR

(400 MHz, DMSO): 12.24 (s, 1H, COOH), 8.23 (d, *J* = 2.3 Hz, 1H, H_k_), 8.00 (dd, *J* = 8.9 Hz, *J* = 2.4 Hz, 1H, H_l_), 7.21 (d, *J* = 10.4 Hz, 1H, H_m_), 7.13 (m, 2H, H_g_, H_e_), 6.87 (d, *J* = 9.0 Hz, 1H, H_j_), 6.80 (t, *J* = 7.3 Hz, 1H, H_f_), 6.66 (d, *J* = 7.8 Hz, 1H, H_d_), 6.00 (d, *J* = 10.4 Hz, 1H, H_i_), 3.5–3.3 (m, 2H, H_c_), 2.62 (m, 2H, H_b_), 1.2 (s, 3H, H_h_), 1.09 (s, 3H, H_h_). The obtained ^1^H NMR spectrum is in agreement with the previously reported spectra.^18–20^

### Liposomes preparation

Liposomes were fabricated by the classical hydration of a thin lipid film method. 1-Palmitoyl-2-oleoyl-*sn*-glycero-3-phosphocholine (POPC) and sphingomyelin (SM) were purchased from NOF Europe Corporation (Germany), cholesterol and 1,3,3-trimethylindolino-6′-nitrobenzopyrylospiran (spiro II) were purchased from Sigma-Aldrich, 1,1′-dioctadecyl-3,3,3′,3′-tetramethylindodicarbocyanine 4-chlorobenzenesulfonate salt (DiD) and 6-dodecanoyl-2-dimethylaminonaphthalene (Laurdan) were purchased from ThermoFisher Scientific. Stock solutions of phospholipids and cholesterol were prepared at a concentration of 1.0 mg mL^−1^ in chloroform or chloroform/methanol (1 : 1) mixture. Stock solutions of DiD and Laurdan were prepared at a concentration of 0.1 mg mL^−1^ in chloroform. Stock solutions of mechanophore molecules (spiro I and II) were prepared at a concentration of 1.0 mg mL^−1^ in tetrahydrofuran (THF). The desired composition, in molar ratio, was achieved by mixing the required volumes these stock solutions (Table S1†). The organic solvent(s) were evaporated under a nitrogen steam and the lipid film was then dried under a vacuumed desiccator overnight. The lipid film was hydrated in Dulbecco's Phosphate Buffered Saline (DPBS, without calcium chloride and magnesium chloride, from Sigma-Aldrich) with total lipid concentration of 5.0 mM in all formulations.

To prepare fluorescein-probes loaded liposomes, the lipid film was rehydrated with 1 mL of 0.1 mg mL^−1^ of the probe, respectively 5-carboxyfluorescein, 4 kDa and 40 kDa fluorescein isothiocyanate–dextran (Sigma-Aldrich) in DPBS. Following rehydration step, the mixture was stirred overnight and then extruded for 21 cycles through a polycarbonate membrane with 0.1 μm pore size (Avanti Mini Extruder) at room temperature. To prepare rapamycin (Alfa Aesar, Thermo Fisher Scientific) loaded liposomes, the lipid film was rehydrated with 1 mL of 5 μM rapamycin in DPBS (diluted from 200 μM stock in ethanol). All the prepared liposomal formulations were passed through a PD10 Desalting Column Filter (GE Healthcare) according to the supplier's gravity protocol, with PBS as elution buffer. For rapamycin loaded formulation, ‘liposome 40% cholesterol’, encapsulation efficiency was 87.1 (±7.0)% and drug loading 0.17% (±0.01%).

### Size distribution measurement and analysis

Liposomes were characterised in terms of particle size distribution by Dynamic Light Scattering (DLS). The mean diameter and particle size distribution of liposome formulations were determined using ZetaSizer 2000 (Malvern Instruments) at 173° backscatter with measurements performed at 25 °C using disposable ZEN0040 cuvettes. Results are the mean (± standard deviation, SD) of three repeats.

### Laurdan generalized polarization (GP)

For membrane fluidity study, liposomes were formulated containing 0.2% molar ratio of Laurdan probe. Fluorescence measurements (excitation and emission spectra) were performed with a Tecan Plate Reader. Fluorescence was excited at 350 nm and emission spectra were recorded between 400 and 600 nm. Measurements were performed before and after sonication (6 minutes using commercially available portable ultrasound unit; specifications: 1 MHz ± 10%, effective radiating area 4 cm^2^ ± 20%, effective intensity 2.4 W cm^−2^) for 100 μL of liposomal suspension (0.5 mM of total lipids) in a 96 well black plate, protected from light by aluminium foil. A thin layer of ultrasonic gel (Anagel) was applied between the plate and the ultrasonic device (Carer Spark). Generalised polarisation (GP) values were calculated by the equation: GP = (*I*_440_ − *I*_490_)/(*I*_440_ + *I*_490_), as determined by Parasassi.^21^ Results are the mean (± standard deviation, SD) of three repeats.

### UV-Vis spectra

UV-Vis absorption spectra were acquired before and after sonication (6 minutes, 1 MHz) for 1 mL of liposomal suspension (0.5 mM of total lipids) in a quartz microcell (path length of 10 mm), protected from light. A thin layer of ultrasonic gel was applied between the bottom of the microcell and the sonicator. Spectra were recorded in the range of 400–700 nm in absorbance mode with a bandwidth of 0.2 nm and a scanning speed of 240 nm min^−1^ using a Beckman UV/Vis spectrophotometer.

### *In vitro* release of fluorescent hydrophilic probes

5-carboxyfluorescein (FITC), and 4 kDa and 40 kDa fluorescein isothiocyanate–dextrans (FITC 4 kDa and FITC 40 kDa) were used as hydrophilic model drugs for release studies. For the ultrasound-triggered experiments, 0.5 mL of liposomal suspension (0.5 mM of total lipids) was placed in a 20 mL glass vial, which was sealed and covered from light by aluminium foil. Ultrasonic gel was then placed between the glass vial and the ultrasonic source. Ultrasound (1 MHz) was applied for 6 minutes. Sonicated liposomal suspensions were transferred immediately into 0.5 mL centrifugal filters (MWCO 100 kDa, Amicon Ultra) and the suspensions centrifuged at 14 000 × *g* for 10 minutes (according to the supplier's protocol). Release from non-sonicated and surfactant (1% Triton X-100)-disrupted liposomes was obtained following the same procedure. After centrifugation, 100 μL of solution from the filtrate collection tube was transferred in 96 well black plate and the fluorescence intensity was recorded by Tecan Plate Reader (*λ*_ex_ 490 nm and *λ*_em_ 535 nm). The release of dye from the liposomes was quantified according to the following equation: release (%) = *F*/*F*_break_ × 100 where *F* is the fluorescence of the solution and *F*_break_ is the fluorescence of the surfactant (Triton X-100)-disrupted liposome solution. No measurable losses observed on ultrafiltration of solutions of probes per sec (Fig. S2†). Data represent the mean (±standard deviation, SD) of three independent experiments.

### *In vitro* release of rapamycin

For the ultrasound-triggered experiments, 0.5 mL of rapamycin loaded liposomes (5 mM of total lipids) was placed in a 20 mL glass vial, which was sealed and covered from light by aluminium foil. Ultrasonic gel was then placed between the glass vial and the ultrasonic source. Ultrasound (1 MHz) was applied for 6 minutes. Sonicated and non-sonicated liposomal suspensions were transferred immediately into 0.5 mL centrifugal filters (MWCO 100 kDa, Amicon Ultra) and the suspensions centrifuged at 14 000 × *g* for 10 minutes (according to the supplier's protocol). After centrifugation, the solution from the filtrate collection tube was transferred into a quartz microcell (path length of 10 mm) and the absorbance intensity was recorded in the range of 240–450 nm (bandwidth of 0.2 nm, scanning speed of 240 nm min^−1^) using a Beckman UV/Vis spectrophotometer. Data shown in ESI (Fig. S3†).

### Cell culture and transfections

CFP-FKBP plasmid, a gift from Tobias Meyer (Addgene plasmid # 20160; https://n2t.net/addgene:20160; RRID:Addgene_20160),^22^ was prepared according to manufacturer's instructions using the NucleoBond® Xtra Midi kit (Machery-Nagel) and quantified *via* spectrophotometry. HeLa CCL2 and Mito-YFP-FRB cell lines (ECACC 15042201) were transfected with linear CFP-FKBP plasmid DNA to obtain, respectively, CFP-FKBP transfected HeLa and CFP-FKBP transfected HeLa Mito-YFP-FRB stable cells lines. CFP-FKBP plasmid DNA was incubated with DraIII-HF® restriction enzyme (30 units of restriction enzyme per 3 μg of plasmid DNA) for 1 h at 37 °C and then purified *via* spin column. Recovered linear CFP-FKBP DNA was transfected using Lipofectamine 3000 (invitrogen) according to the manufacturer's instructions. These cells were allowed to recover for 48 hours before being subject to antibiotic selection for 14 days. The resulting stable cell lines were routinely maintained in Dulbecco's Modified Eagles Medium (DMEM + GlutaMAXTM, Gibco, Life technologies) supplemented with 10% v/v Foetal Bovine Serum (FBS) and the required selection antibiotics: 100 μg mL^−1^ of hygromycin B (Sigma-Aldrich) for Mito-YFP-FRB cell lines; 300 μg mL^−1^ G 418 disulfate salt (Sigma-Aldrich) CFP-FKBP transfected HeLa; 100 μg mL^−1^ of hygromycin B and 300 μg mL^−1^ G 418 disulfate salt for CFP-FKBP transfected HeLa Mito-YFP-FRB cells, 20 μg mL^−1^ gentamicin for HeLa CCl_2_. The cells were maintained in a 100 mm cell culture dishes (Sarstedt) at 37 °C and 5% CO_2_. The medium was changed three times a week and cells passaged before reaching confluency.

### Fluorescence microscopy and flow cytometry (FRET)

Cells were seeded at a density of 1 × 10^5^ cells per mL in a 12 well plate and incubated at 37 °C for 48 hours in 1 mL of the appropriate growth medium. CFP-FKBP transfected HeLa Mito-YFP-FRB cells were treated with either 1 mL of ‘liposome 40% cholesterol’ or ‘liposome 40% cholesterol + spiro I′ (at 0.5 mM of total lipid concentration in HEPES-buffered HBSS, loaded with ∼500 nM of rapamycin), 500 nM free rapamycin, or HEPES-buffered HBSS buffer. The cells were incubated for 2 hours at 37 °C. After this time, the liposomal suspension was removed and cells were thoroughly washed 5 times with PBS. For the cells exposed to ultrasound, a thin layer of ultrasonic gel was applied between the plate and the ultrasonic device, and the cells were sonicated for 6 minutes.

For fluorescence microscopy, cells were grown on coverslips and following treatment fixed with 4% formaldehyde. Coverslips were mounted on microscope slides, and imaged with an Olympus BX51 microscope equipped and Retiga R1 CCD camera (Qimaging). All images of fluorescent proteins were captured at equal exposure settings without prior illumination. Image acquisition was controlled by μManager open-source software.

For flow cytometry measurements, the cells were detached from the plates by 5 min incubation at 37 °C with Accutase (Sigma-Aldrich) following supplier's protocol. Cell suspension obtained was centrifuged, fixed with 4% formaldehyde, washed with glycine buffer (0.1 mM in PBS), and re-suspended in PBS. All samples were constantly protected from light by aluminium foil. FRET and DiD fluorescence was acquired using a MoFlo Astrios EQ flow cytometer (Beckman Coulter) equipped with 405 nm, 488 nm and 640 nm lasers. To measure CFP and FRET, cells were excited with the 405 nm laser and fluorescence was collected in the CFP channel with a standard 448/59 filter, while the FRET-signal was measured with a 529/28 filter. To measure YFP, cells were excited with the 488 nm laser while emission was read with a 530/40 filter. At least 10^4^ events/sample were acquired and analysed using Weasel Software version 3.5. Gating strategies are illustrated in ESI, Fig. S4 and S5.†

## Results and discussion

Particle size analysis in Fig. 1B demonstrates a relatively narrow particle size distribution of fabricated liposomal formulations (Table S2†), including liposomes with membrane embedded mechanoresponsive spiropyran molecules. The average particle sizes for different systems are between 130 to 180 nm (ESI Fig. S6†) – the particle size appropriate for endocytosis by cells.^14,23^ The particle size distribution profiles indicate that incorporation of 20 mol% of mechanoresponsive spiro I or II compounds (Fig. 1B) into lipids composition does not appreciably influence the liposome average particle size and size distribution. It should be noticed that the molecular size of the mechanoresponsive compounds (MW 380 and 320 Da, respectively) is comparable to the size of cholesterol (386 Da) used in liposome fabrication, although their ring structure is less planar relative to cholesterol. The data further show that exposure of mechanoresponsive-liposomes to ultrasound at 1 MHz does not have a profound effect on the particle size distribution and scattering profiles, indicating that the conditions of exposure do not lead to a considerable loss of liposomes integrity.

**Fig. 2:**
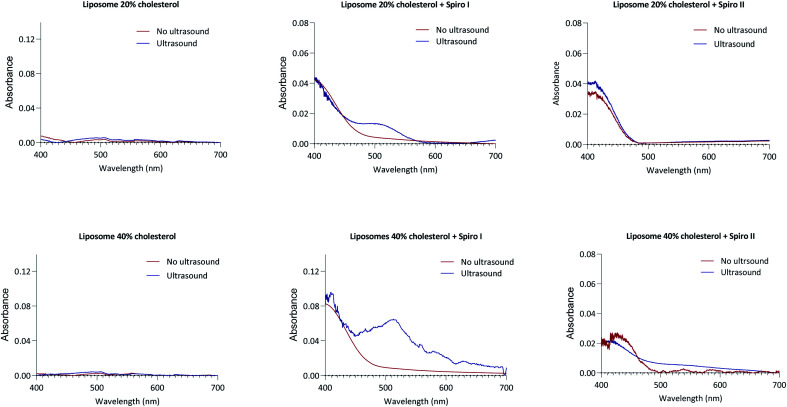
UV-Vis spectrophotometry analysis of liposomes and mechanoresponsive-liposomes prior and following ultrasonication. Spectra shown for ‘control’ and liposomes with 20 and 40 mol% content of cholesterol with incorporated either spiro I or spiro II compounds. Suspension of liposomes exposed to 1 MHz ultrasonication for 6 minutes and UV-Vis analysis performed immediately following exposure.

The UV-Vis absorption profiles for liposomes formulations prepared with spiro I and II compounds are shown in Fig. 2. These compounds are practically insoluble in water whereby commercially available spiro II, and its open merocyanine form, were shown both insoluble and not exhibiting UV-Vis absorption in water.^24^ Therefore absorption observed (Fig. 2) can be attributed to ‘solubilised’ molecules embedded in liposomal membrane. Dissolved in organic solvents, spiropyran type structures show absorption band at around 380 nm;^25^ similar is indicated in Fig. 2 with a peak in region below 400 nm. The presence of this peak therefore indicates incorporation of spiropyran-compounds into liposomal membrane. UV-Vis spectra in Fig. 2 were recorded immediately following exposure of spiropyran–liposomes to 1 MHz sonication and they indicate appearance of an open merocyanine form of spiropyran molecule for spiro I, as judged from an absorption peak around 510 nm region, not observed in the spectra of spiropyran–liposomes not exposed to ultrasound, or ‘control’ liposomes. It should be noticed that spiropyran-based molecules are solvatochromic,^25,26^ hence the position of absorption maximum is influenced by a solvent which, in this case, may be extended to the local environment provided by liposomal lipid membrane.

Considering spiro II, the reasons for lack of absorption peak for merocyanine form of spiro II in liposomal formulations following exposure to 1 MHz ultrasound are not clear at this stage. Possible contribution to the observed difference in spiro I and II behaviour on ultrasound exposure may be arising from differences in their molecular structures. Spiro I contains carboxyethyl functionality on nitrogen of indole ring, rather than methyl present in spiro II, making spiro I molecule relatively polar (TPSA for Spiro I is 95.6 Å^2^, and for spiro II 58.3 Å^2^). Spiro I may hence be inserted at the inter-/surface of the lipid bilayer, less ‘deep’ in the bilayer than less polar spiro II molecule, and may consequently be relatively more exposed to mechanical stress created to the lipid bilayer inter-/surface by ultrasound treatment. In addition to above mentioned factors, it may also reflect the observation that solutions of this compound in solvents of low polarity (such as would be expected from environment created ‘deep’ in a lipid bilayer of liposomal membrane) are colourless.^26^

**Fig. 3:**
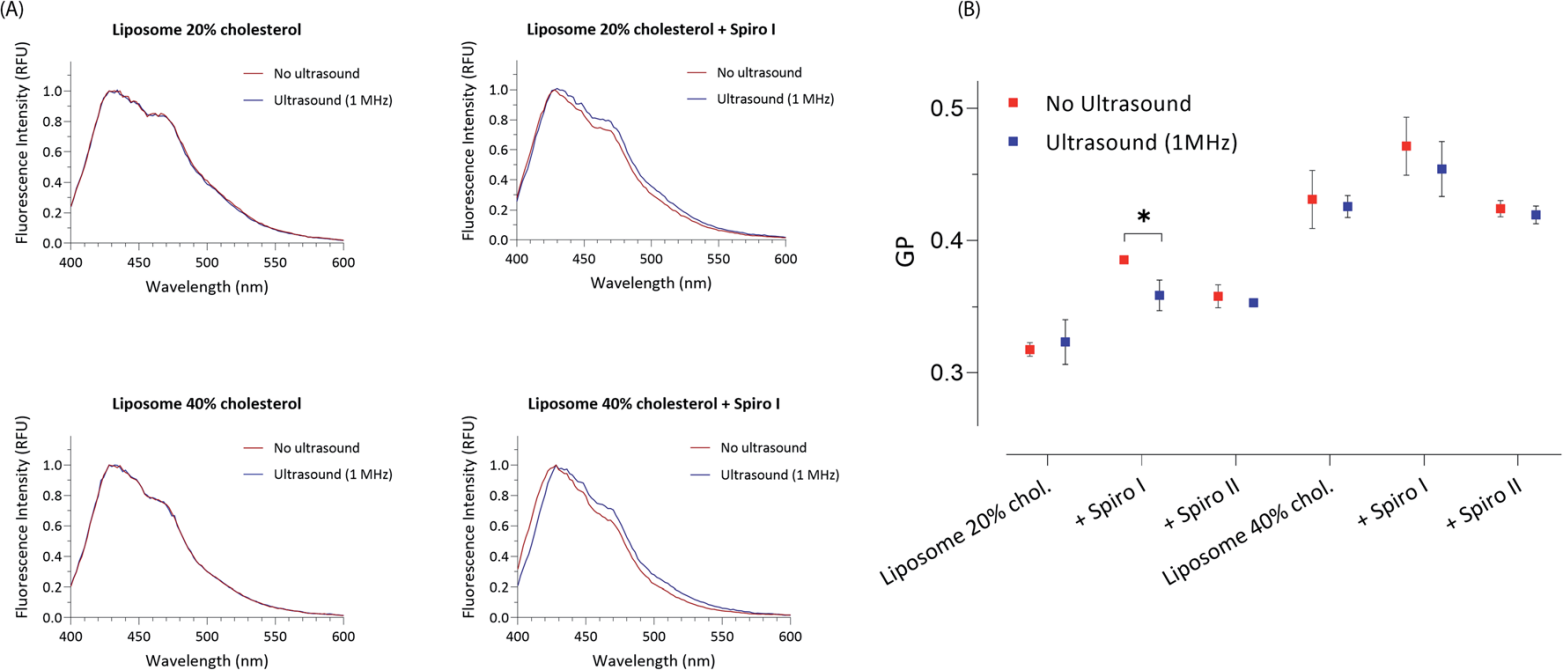
Laurdan generalised polarisation (GP) analysis of mechanoresponsive-liposomes with or without exposure to ultrasound; illustrative Laurdan emission profiles for spiro I (A), and calculated GP ratios (B), *P < 0.05 by t-test.

Profiles shown in Fig. 3A depict changes in polarisation of Laurdan fluorescent probe; Laurdan is a ‘classical’ membrane fluidity sensing probe and its emission spectrum reveals state of lipid bilayer polarity/order; emission maximum at 440 nm corresponds to a more ordered, and maximum at 490 nm to a more disordered lipid bilayer.^27^ The profiles illustrate that exposure to ultrasound of mechanoresponsive spiro I-liposomes causes a noticeable change in Laurdan emission spectrum; an increase in intensity of 490 nm maximum indicating changes in the membrane towards liquid disordered structure for both 20 and 40 mol% cholesterol content. This effect is not seen for exposure to ultrasound of ‘control’ liposomes. Calculated general polarisation values (GP) for all tested systems in Fig. 3B indicate more pronounced effect of embedding spiro I, relative to spiro II, into liposomal membrane. This reflects observations from Fig. 2.

It should be also noticed that GP values show a clear difference in fluidity of membrane for 20 and 40 mol% cholesterol containing liposomes – with higher GP values for 40 mol% cholesterol liposomes indicating existence of a membrane (lipid bilayer) with higher order; values of GP around 0.3 and below are generally accepted to indicate a membrane in a fluid state, whilst values above 0.3 indicate ordered, gel state of lipid bilayer membranes.^28,29^ Interesting observation is that incorporation of spiropyran molecules into liposomes with 20 mol% content of cholesterol (on expense of mol% of POPC) increases liquid order structure of the membrane, as reported by higher GP values (Fig. 3B). This effect appears less prominent in 40 mol% cholesterol membrane, already in the state of higher level of liquid order. This again points to a conclusion that the mechanoresponsive compounds are embedded in lipid bilayer membrane of liposomes.

**Fig. 4:**
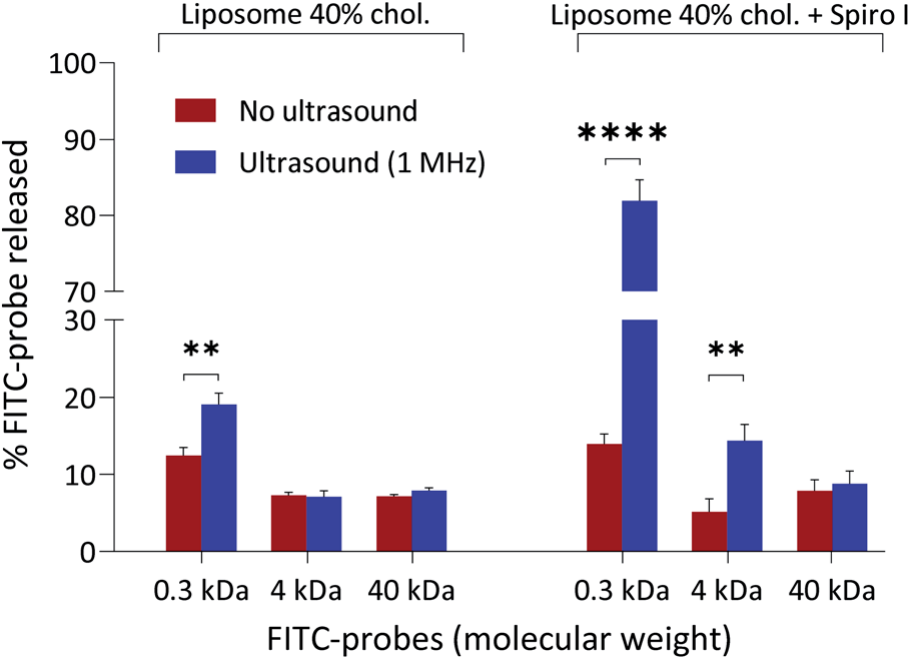
Ultrasound-triggered release of model compounds from mechanoresponsive-liposomes. Release of encapsulated model hydrophilic compounds from ‘control’ and mechanoresponsive-liposomes with or without exposure to ultrasound. Data expressed as % release of Triton X-100 surfactant treated liposomes taken as 100%. **P < 0.005, ****P < 0.0001 by t-test.

The release data for encapsulated hydrophilic probes from fabricated liposomes are summarised in Fig. 4. The selection of molecular probes used here reflects typical sizes of small molecular weight drugs (∼0.5 kDa), peptides and small proteins (*e.g.* insulin 5.808 kDa), and macromolecular biologics (antibody fragments ∼25 kDa). The results point to the following conclusions: (i) exposure to ultrasound of ‘control’ liposomes (without embedded spiropyran) does not cause a profound release of encapsulated hydrophilic probes; the highest release is observed for low molecular weight 0.3 kDa FITC and amounts to 19%, (ii) exposure of mechanoresponsive-liposomes with embedded spiropyran to ultrasound appears to result in a size-dependent release of encapsulated hydrophilic probes: 6-fold increase in FITC 0.3 kDa release (amounting to approximately 80% release), 2.5-fold increase in FITC-Dextran 4 kDa (approximately 15% release), and no significant increase in release of 40 kDa FITC-dextran. These results were obtained following a single exposure of liposomes to 1 MHz for 6 minutes and immediate analysis. Molecular dimensions of 4 kDa and 40 kDa FITC-dextran probes are around 2.8 and 9 nm, respectively.^30^ Absence of enhanced release of 40 kDa probe on sonication at 1 MHz of mechanoresponsive-liposomes indicates that these conditions do not cause membrane perturbations/pores formation which would allow passage of 9 nm sized species, or a total loss of liposomes integrity that would result in a complete release of the encapsulated probe (as is the case on treatment with Triton X-100 surfactant). The latter corroborates with particle size analysis in Fig. 1B, which does not point to a significant effect of ultrasound in experimental conditions applied on particle size distribution profiles and scattering intensity of mechanoresponsive-liposomes. Enhanced release of low and medium molecular weight probes (0.3 kDa FITC and 4 kDa FITC-dextran) from mechanoresponsive liposomes exposed to ultrasound treatment would agree with observed increase in liquid disorder of liposomal membranes on exposure to ultrasound (Fig. 3).

**Fig. 5:**
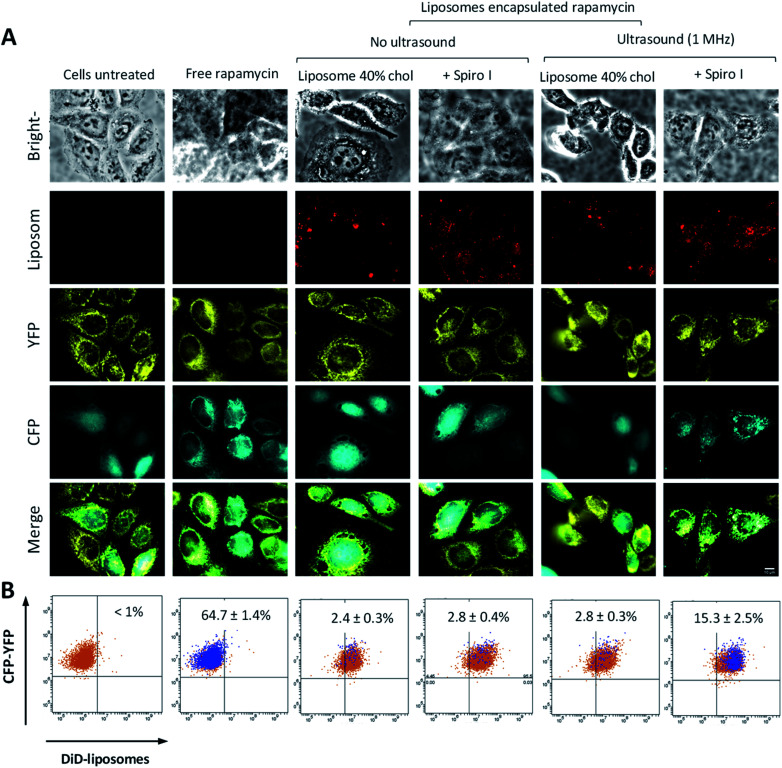
(A) Fluorescence microscopy of reporter cell system co-expressing α-CFP-FKBP (cyan) and YFP-FRB (yellow) following exposure to different treatments, as indicated above the images; ‘ultrasound’ denotes that cells were exposed to 1 MHz ultrasound continuously for 6 minutes. (B) Flow cytometry dot plots of FRET effect for reporter cell system following different treatments – these correspond to microscopy image above. Orange dots above the horizontal line positioned in upper left quadrant indicate cells showing fluorescence from α-CFP-FKBP and Mito-YFP-FRB, blue dots above the horizontal line positioned in upper left quadrant indicate cells showing FRET from α-CFP-FKBP and YFP-FRB pair, orange dots above the horizontal line and positioned right of vertical line in right upper quadrant indicate cells showing both α-CFP-FKBP and Mito-YFP-FRB fluorescence as well as internalization of DiD labelled liposomes, ‘overlap’ of orange and blue dots in upper right quadrant indicates cells that show both internalization of liposomes and FRET effect of CFP-FKBP and YFP-FRB pair driven by the presence of free rapamycin in the cytoplasm. Percentage of cells in population showing FRET is reported in each dot plot. The flow cytometry gating strategy illustrated in ESI.†

To assess intracellular release of liposomes encapsulated cargo, we developed a reporter cell system based on HeLa-Mitotrap cells. The transfected cells co-express (i) a mitochondrial trapping construct YFP-FRB and (ii) cytosolic α-FKBP domain-CFP. Such cloned cells create a reporter system which, on binding of mitochondrial YFP-FRB (yellow) with cytosolic α-CFP-FKBP (cyan), driven by the intracellular presence of free rapamycin, show redistribution of cytosolic α-CFP-FKBP protein towards mitochondria and its co-localisation with YFP-FRB (visualized by microscopy, Fig. 5A ‘free rapamycin’), as well as creating a FRET effect between CFP donor and YFP acceptor pair (measured by flow cytometry, Fig. 5B and ESI Fig. S4 and S5†). Cytosolic presence of rapamycin induces dimerisation of FKBP rapamycin binding domain with rapamycin binding protein (FRB) on the mitochondrial surface. Considering images in Fig. 5A, untreated cells show (i) perinuclear distribution of yellow puncta, indicative of mitochondria associated YFP-FRB, and (ii) smeared cytosolic distribution of cyan colour of α-CFP-FKBP. Application of rapamycin (‘free rapamycin’) results in changes in distribution pattern of cyan α-CFP-FKBP in cytoplasm to coincides with yellow YFP-FRB, indicating redistribution of cytosolic α-CFP-FKBP to mitochondrial surface and its co-localisation with YFP-FRB, confirming functioning of the cell reporter system. Regarding cells treated with rapamycin encapsulated into liposomes, either with or without ultrasound exposure, or mechanoresponsive liposomes without exposure to ultrasound these depict (i) intracellular distribution of red puncta, indicative of liposomes associated DiD fluorescence, in addition to (ii) perinuclear distribution of yellow puncta, indicative of mitochondria associated YFP-FRB and (iii) smeared cytosolic distribution of cyan colour of α-CFP-FKBP, as seen for untreated cells. The images hence indicate that these systems do not provide sufficient level of free cytoplasmic rapamycin to observe the fluorescent proteins co-localisation, even after exposure to ultrasound of liposomes without embedded spiro-compound. On treatment with rapamycin loaded mechanoresponsive liposomes and exposure to ultrasound a redistribution of cyan α-CFP-FKBP protein and its co-localization with yellow YFP-FRB can be observed. This demonstrates that mechanoresponsive-liposomes deliver and release intracellularly on external ultrasound stimulus a sufficient amount of free rapamycin for the fluorescent proteins co-localization to be observed.

It should be also noticed from the brightfield images that morphology of the cells does not indicate observable negative effects of exposure to mechanoresponsive-liposomes, with or without subsequent 1 MHz sonication, as judged from a comparison to morphology of control, untreated cells.

Further analysis applying flow cytometry to quantify a α-CFP-FKBP (donor) and YFP-FRB (acceptor) pair FRET effect (dot plots in Fig. 5B) illustrates the presence of FRET in a sub-population of approximately 64% of cells treated with free rapamycin (positive control), again confirming functioning of the cell reporter system. Considering treatments with rapamycin loaded ‘control’ liposomes, these are taken by the reporter cells (appearing in upper right quadrant) but there is no significant FRET effect whether ultrasound was applied or not, indicating insufficient presence of free rapamycin in the cell cytosol to create measurable FRET effect of donor–acceptor proteins. A FRET effect in approximately 15% of cells is evident on the treatment with mechanoresponsive-liposomes and application of 1 MHz ultrasound, demonstrating the intracellular release of rapamycin from such liposomes on external ultrasound stimulus.

## Conclusions

This study set out to test if liposomal system can be designed such that a control over intracellular release of loaded drug can be achieved by applying external ultrasound stimulus – at 1 MHz ultrasound frequency applied in clinical setting. We hence embedded mechanoresponsive spiropyran-based compound into liposomal lipid bilayer. The results demonstrate that following internalisation of such designed mechanoresponsive-liposomes by cells, encapsulated drug is released intracellularly on external ultrasound application, which is attributable to opening of spiropyran molecule caused by exposure of liposomes to ultrasound. The study hence indicates feasibility of a concept where intracellular drug release from nano-sized mechanoresponsive liposomes can be triggered on demand by external ultrasound stimulus.

## Author contributions

RC conceptualization, investigation, writing; DO methodology; ER methodology, MZ supervision, conceptualization; GM supervision, conceptualization; AH methodology, supervision; SS conceptualization, supervision, writing.

## Conflicts of interest

There are no conflicts to declare.

## Supplementary Material

RA-012-D2RA02307E-s001
