# Supplementary material for: A mechanoresponsive nano-sized carrier achieves intracellular release of drug on external ultrasound stimulus
Source: RSC Adv. 2022 Jun 6;12(26):16561–9. doi: 10.1039/d2ra02307e (PMC9169073; doi:10.1039/d2ra02307e)
Supplement: RA-012-D2RA02307E-s001 [file RA-012-D2RA02307E-s001.pdf]

Electronic Supplementary Material (ESI) for RSC Advances.
This journal is © The Royal Society of Chemistry 2022

Supporting information

Mechanoresponsive nano-sized carrier achieves intracellular delivery and release of drug on external ultrasound stimulus

Rosa Catania[a,b], David Onion[b], Emanuele Russo[a], Mischa Zelzer[a], Giuseppe Mantovani[a], Alan Huett[b], and Snow Stolnik[a,]*

[a] School of Pharmacy, University of Nottingham, Nottingham NG7 2RD, United Kingdom; [b] School of Life Sciences, University of Nottingham, Nottingham NG7 2UH, United Kingdom.

* Snow Stolnik, snow.stolnik@nottingham.ac.uk

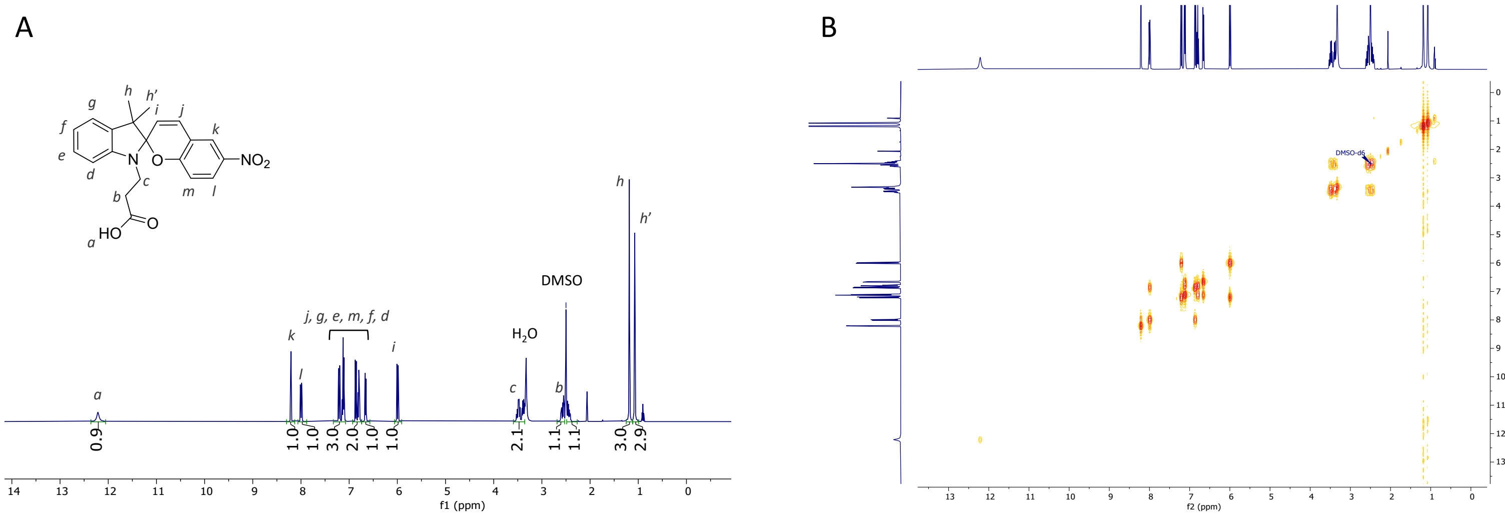

Figure S1. ^{1}H NMR spectrum (A) and COSY NMR spectrum (B) of Spiropyran I in DMSO-d6.

Table S1 Lipid composition of liposome formulations used in the study.

	% Molar ratio				
**Formulation**	**POPC**	**SM**	**Cholesterol**	**Mechanophore**	**DiD or Laurdan***
Lipo 20%	65.25	14.55	20.00	0.00	0.2
Spiro I 20%	45.25	14.55	20.00	20.00	0.2
Spiro II 20%	45.25	14.55	20.00	20.00	0.2
Lipo 40%	45.26	14.55	40.00	0.00	0.2
Spiro I 40%	25.25	14.55	40.00	20.00	0.2
Spiro II 40%	25.25	14.55	40.00	20.00	0.2

POPC: palmitoyl-2-oleoyl-sn-glycero-3-phosphocholine; SM: sphingomyelin, Chol: cholesterol: Mechanophore is compound 2 for Spiro I formulation, and commercial compound 1′,3′-Dihydro-1′,3′,3′-trimethyl-6-nitrospiro[2H-1-benzopyran-2,2′-(2H)-indole] (Sigma-Aldrich) for Spiro II formulations. * Depending from the study, 0.2% mol ratio of DiD or Laurdan was added.

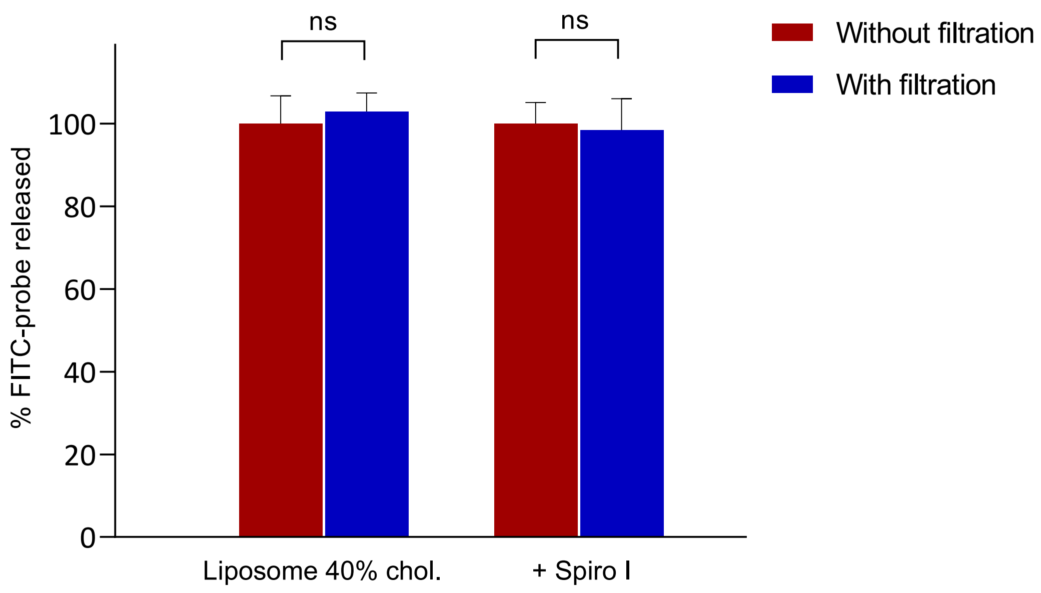

Figure S2. Percentage of FITC-probe release content after treatment with Triton X-100 with or without filtration.

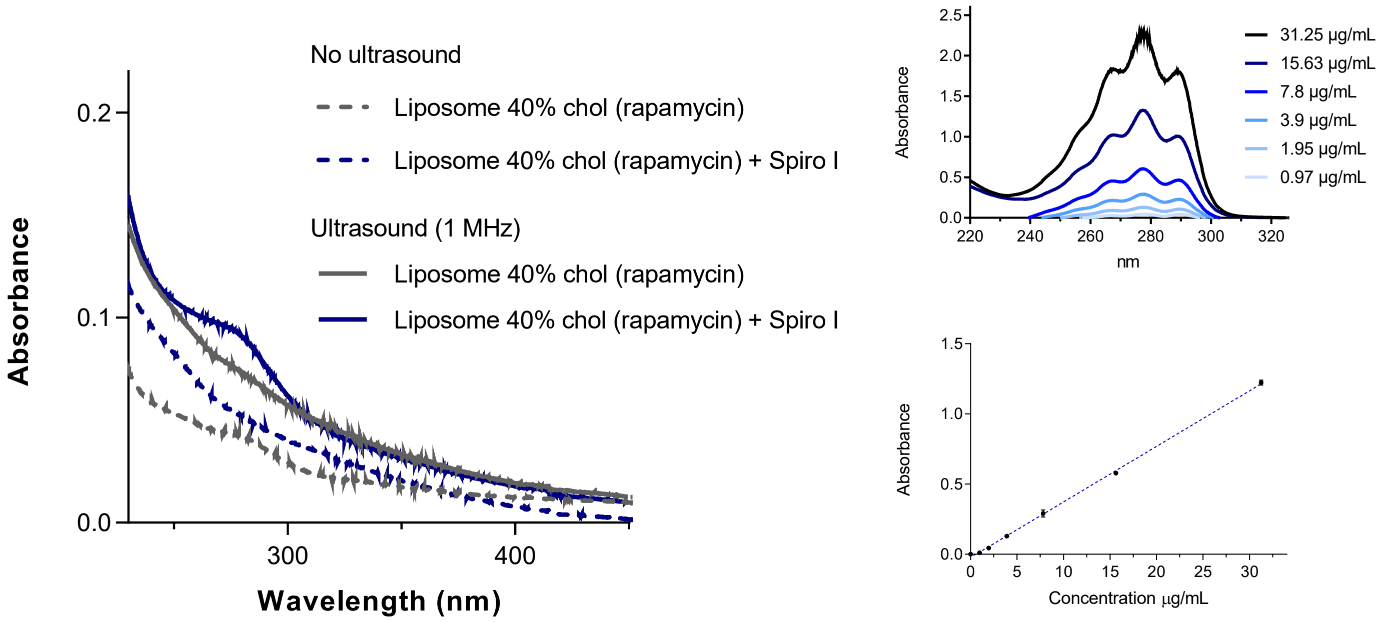

Figure S3. UV-Vis absorbance profiles of media for rapamycin release from different liposomal formulations, with and without exposure to ultrasound. Experimental protocol described in main text. Profiles show the presence of peak at 280 nm for rapamycin released into medium from liposomes ('Liposome rapamycin') and from mechanoresponsive-liposomes ('liposome rapamycin + Spiro I + ultrasound') following exposure to 1 MHz ultrasound continuously for 6 minutes. Relevant calibration curve shown. No measurable loss during the ultrafiltration was observed.

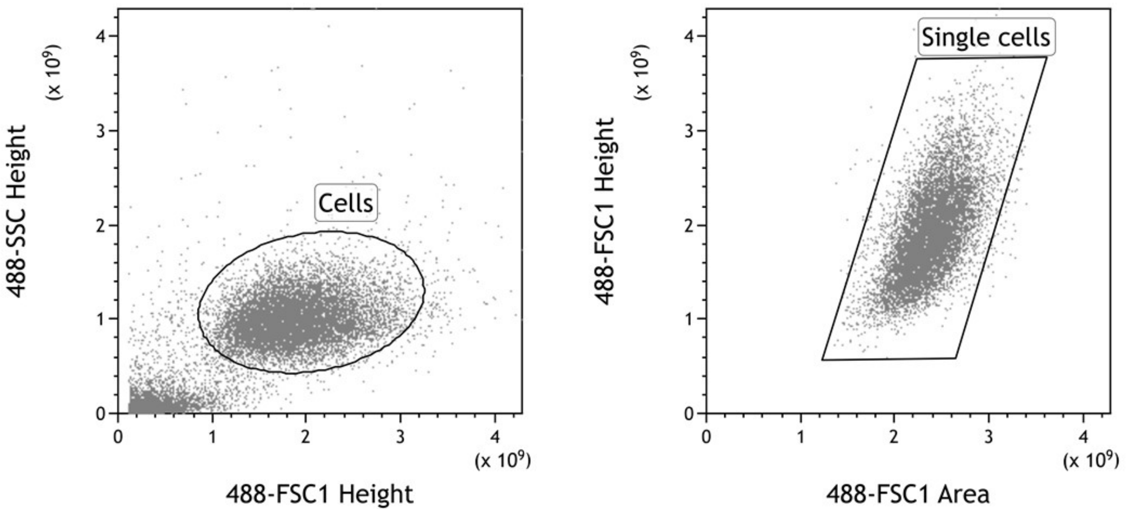

Figure S4. Flow cytometry gating strategy applied to cells according to forward and sideward scatter (FSC/SSC). Cells were then gated in an FSC-Height (FSC-H) and FSC-Area (FSC-A) dot plot to eliminate doublets.

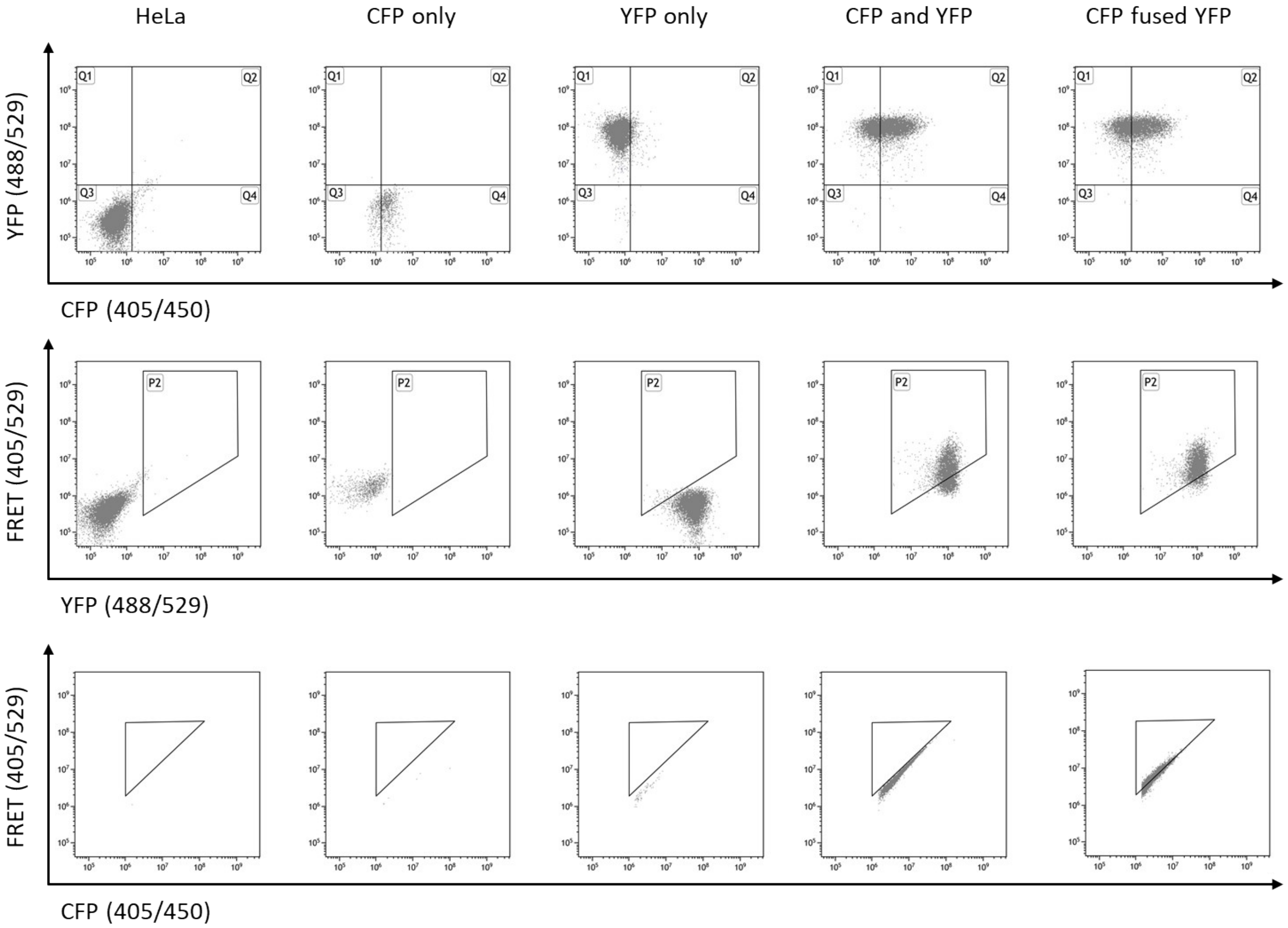

Figure S5. Flow cytometry FRET gating strategy. Analysis of HeLa cells and transfected cells expressing reporter FRB-YFP and cytosolic α-FKBP domain-CFP system; measured are: CFP only, YFP only, CFP and YFP, and CFP-YFP dimerised ('fused') proteins. Analysis performed on MoFlo Astrios EQ flow cytometer (Beckman Coulter). Double positive cells were gated (panel 1) and false positive FRET signals resulting from YFP

excitation by the 405 nm laser were excluded (panel 2). The remaining cells were evaluated for FRET by adjusting a gate for cells which are expressing both CFP and YFP (but not dimerised) and should thus be FRET-negative (panel 3).

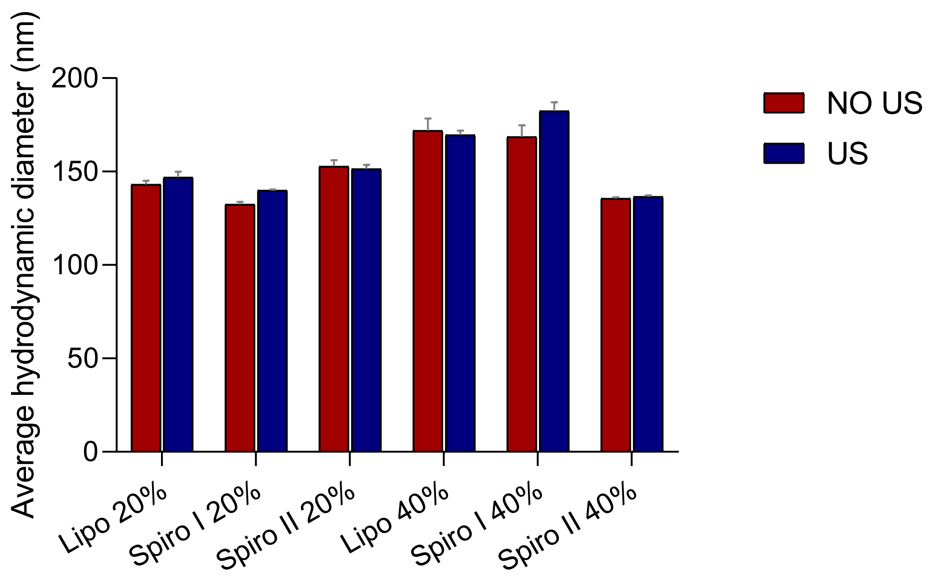

Figure S6. Hydrodynamic average diameter of liposome formulations obtained from dynamic light scattering measurements.

Table S2 Polydispersity index (PdI) for the size distributions data shown in Fig. 1B and S6.

	**No ultrasound**		**Ultrasound (1 MHz)**	
**Formulation**	**PdI**	**S.D.**	**PdI**	**S.D.**
Lipo 20%	0.21	0.01	0.25	0.02
Spiro I 20%	0.13	0.02	0.16	0.02
Spiro II 20%	0.28	0.01	0.26	0.01
Lipo 40%	0.30	0.01	0.31	0.02
Spiro I 40%	0.21	0.02	0.25	0.02
Spiro II 40%	0.18	0.01	0.17	0.01

S.D. indicates standard deviation
